# Supplementary material for: Moisture adsorption-desorption full cycle power generation
Source: Nat Commun. 2022 May 9;13:2524. doi: 10.1038/s41467-022-30156-3 (PMC9085775; doi:10.1038/s41467-022-30156-3)
Supplement: Supplementary file 1 — Supplementary Information [file 41467_2022_30156_MOESM1_ESM.pdf]

Supplementary Information

Moisture adsorption-desorption full cycle power generation

Haiyan Wang[1], Tiancheng He[2], Xuanzhang Hao[2], Yaxin Huang[2], Houze Yao[2], Feng Liu[3]*, Huhu Cheng[1]* & Liangti Qu[1,2]*

[1]Key Laboratory of Organic Optoelectronics & Molecular Engineering, Ministry of Education, Department of Chemistry, Tsinghua University, Beijing 100084, P. R. China.

[2]State Key Laboratory of Tribology, Department of Mechanical Engineering, Tsinghua University, Beijing 100084, P. R. China.

[3]State Key Laboratory of Nonlinear Mechanics, Institute of Mechanics, Chinese Academy of Sciences, Beijing 100190, China.

First principle calculation for chemical shifts

First principle calculation is directly used to study chemical shifts variation with number of water molecules. Different Na^+ ion is surrounded by different number of H_2O molecules with fixed distance (0.22 nm between O atom and Na^+) and orientation. The more realistic atomic configuration at 300 K for $Na+/nH_2O$ could be obtained by molecular dynamic simulations, and the average number (n) of neighbor water molecules within 0.25 nm cutoff is given by statistics (to be specific, 3.91 for $Na+/4H_2O$, 4.06 for $Na+/6H_2O$, 4.67 for $Na+/12H_2O$, and 4.56 for $Na+/24H_2O$), according to which their chemical shifts could be obtained by interpolating first principle calculation.

Molecular dynamic (MD) simulation details for hydration energy of Na^+ ion

To calculate the hydration energy of Na^+ ions with different number of water molecules adhering on graphene, potentials need to be defined first. Here interaction among water molecules is described by SPC model[1], and ions interact only with oxygen atoms of water molecules, and its potential form is given by:

$$E = \frac{1}{4\pi\varepsilon_0}\sum_{i=O}\frac{Z_i Z_m}{r_{im}} + \sum_{i=O} 4\varepsilon\left[\left(\frac{\sigma}{r_{im}}\right)^{12} - \left(\frac{\sigma}{r_{im}}\right)^{6}\right] \quad (1)$$

where *m* and *i* represents ion and the oxygen atom in the ith water molecule, respectively, and ε_0 and Z_i stand for vacuum dielectric constant and the charge of ith atom. Besides, ε and σ determine the magnitude and equilibrium distance of Lennard–Jones potential. This energy form contains two terms. The first term coulomb potential represents charges' interaction, and

the second term Lennard–Jones potential is used to describe the interaction between ion and oxygen atoms. For Na^+, the parameters ($\varepsilon = 0.00208$ eV and $\sigma = 2.85$ Å) could be obtained directly by the previous report[2].

The adhesion between hydrated ion and monolayer graphene is considered by using Lennard–Jones potential the interaction between carbon atoms and oxygen atoms[3], where $\varepsilon = 0.00406$ eV and $\sigma = 3.19$ Å. For monolayer graphene itself, harmonic potential $E_{bond} = K_{CC}(r - r_0)^2$ is used to describe the nearest neighbor carbon atoms' interaction[4], where $K_{CC} = 20.75$ eV/Å^2 and $r_0 = 1.42$ Å. Lennard–Jones potential is used to depict van der Waals interaction among non-nearest neighbor carbon atoms[5], where $\varepsilon = 0.00284$ eV and $\sigma = 3.4$ Å. The interaction cut off is set to 9 Å for all Molecule dynamics simulations.

Large-scale Atomic/Molecular Massively Parallel Simulator (LAMMPS)[6] is chosen to perform MD simulations under *NVT* ensemble at 300 K, and periodic boundary condition is applied here. The simulation box is nearly cubic with its size close to 100 Å. The length and width could slightly deviate from this value since they should be multiple times of the graphene lattice constant in corresponding direction. Time step takes 0.5 fs and the relaxation process lasts for 100 ps (2×10^5 time steps), which could guarantee the entire enter into an equilibrium state. Then in the following 500 ps (10^6 time steps), the Na^+ ion hydration is measured and taken average to obtain their mean value. During these simulations, graphene is frozen to reduce thermal fluctuation so that the Na^+ ion hydration energy could be accurately extracted. There are dissociated free Na^+ ions and boned Na atom anchored on the polymer chains in the system. As the major concern in this work is power generation, only dissociated Na^+ ions are

considered in MD simulations. Besides, our work only studies the balance voltage (that is the voltage at the steady state, in which all physical quantities become time independent) variation with different dissociated free Na^+ ions concentration, thus many complicated dynamic processes (for instance, anchored Na atoms could be hydrated and become dissociated free Na^+ ions at some points and its reverse process) are unnecessary to take into account.

MD Simulation details for graphene foam structure

In our calculation model, each graphene sheet is composed by 7 × 7 coarse grain particles square lattice, SiO_2 fibers are approximated as chains and each of them is made up by 12 coarse grain particles. Here coarse grain size is set to 0.75 μm suggesting graphene sheet is 4.5 μm and SiO_2 fiber is 8.25 μm in length.

The total energy form of graphene[7] is written as:

$$\phi_{System} = \phi_T + \phi_\varphi + \phi_\theta + \phi_{vdw} \tag{2}$$

where the first three terms share same harmonic potential style and are given by:

$$\phi_T = \frac{1}{2} K_T (r - r_0)^2 \tag{3}$$

$$\phi_\varphi = \frac{1}{2} K_\varphi (\varphi - \varphi_0)^2 \tag{4}$$

$$\phi_\theta = \frac{1}{2} K_\theta (\theta - \theta_0)^2 \tag{5}$$

where the spring coefficient of bonds K_T = 16214000 pg·μs^{-2}, and the spring coefficients of two bond angles are K_φ = 527625 pg·μm^2·μs^{-2} and K_θ = 1126 pg·μm^2·μs^{-2}, respectively. r_0 = 0.75 μm, φ_0 = 90°, and θ_0 = 180°, which defines the equilibrium bond distance and angles, respectively. Fibers use same parameters, except they do not have interlayer bond angle φ.

Among different graphene sheets and fibers, Lennard-Jones potential is used to describe van der Waals interaction:

$$\phi_{vdw} = 4\varepsilon[(\sigma/r)^{12} - (\sigma/r)^{6}] \quad (6)$$

where $\varepsilon = 520$ pg· μ m^{2}· μ s^{-2} and the equilibrium distance $\sigma = 0.1$ μm. Graphene sheets with number of 4300 and fibers with number of 4300 are placed in a 30 × 30 × 30 μm^{3} periodic simulation box. With NVT ensemble at 300 K, the system is relaxed until less than 0.1% energy variation within 1000 time steps (time step is 0.5 ps), which outputs a stable composite structure and would be taken as the skeleton for the simulation of hydrated Na^{+} ion diffusion based on kinetic Monte Carlo method.

Kinetic Monte Carlo simulation

N-Fold Way (NFW) algorithm is applied to perform kinetic Monte Carlo simulations. The details about this kinetic Monte Carlo and NFW algorithm could be found in previous reports[8–10]. Here the prepared graphene\fiber composite is considered as a rigid skeleton, hydrated cations cluster could hop to neighbor sites if distance is no more than 0.5 μm. The rate of a diffusional hop is:

$$r_D = \begin{cases} D_0 & \Delta E \leq 0 \\ D_0 exp\left(-\frac{\Delta E}{k_B T}\right) & \Delta E > 0 \end{cases} \quad (7)$$

where ΔE represents energy change induced by hop. The total energy of our model is given by:

$$E_{tot} = \alpha \sum_{i=ions} \frac{Z_i Z_j}{r_{ij} + d_c} + \beta \cdot I_m \quad (8)$$

where the first term is used to describe the interaction between ions, and α is a scaling factor. Its only difference from coulomb potential comes from d_c, which is set to guarantee that the coulomb attraction between cations and anions when they meet on one site is small enough to be broken by kinetic energy, i.e. k_BT. Second term represents linear moisture intensity dependence of ion hydration energy, β is a coefficient to control its magnitude. Note that I_m is the normalized moisture intensity whose value could not be greater than one. The values of d_c, α, and β used in our simulation are set to 1 μm, 0.01 eV • μ m, and 0.1 eV (when $I_m = 1$), respectively.

Characterization

Scanning electron microscope (SEM) were acquired by using a Sirion-200 field-emission scanning electron microscope (FEI Corporation, USA). Energy dispersive X-ray spectra were carried out by the use of an INCAEnergy EDS System (Oxford Instruments, UK). Transmission electron microscope (TEM) were carried out on JEM-100Plus (JEOL, Japan). The dynamic gravimetric vapor sorption (DVS) was taken out by DVS-1000 instrument (Surface Measurements Systems, UK). Solid state NMR nuclear magnetic resonance (NMR) tests were performed using JNM-ECZ600R (JEOL, Japan). Kelvin probe force microscopy (KPFM) was recorded on a Brucker Dimension Icon machine. Fourier transform infrared (FTIR) spectra were conducted on a Thermo IS5 machine (Thermo Nicolet, USA). X-ray photoelectron spectra (XPS) were taken on an ESCALAB 250 photoelectron spectrometer (Thermo Fisher) with an Al Kα source. Zeta potentials were measured by using a Surpass zeta potential analyzer

(Anton Paar, Austria). Optical images were collected from Zeiss Axio Scope.A1 optical microscope. The EIS measurements were performed on a Solartron 1470E electrochemical workstation (Solartron Analytical). Confocal laser scanning microscope (CLSM) were performed using BrukerOpterra II (Bruker Nano Surfaces). Mercury intrusion porosimetry (MIP) experiment was measured by using Autopore IV 9500 (Micromeritics). The mechanical test was conducted by an Instron 5943 universal testing machine.

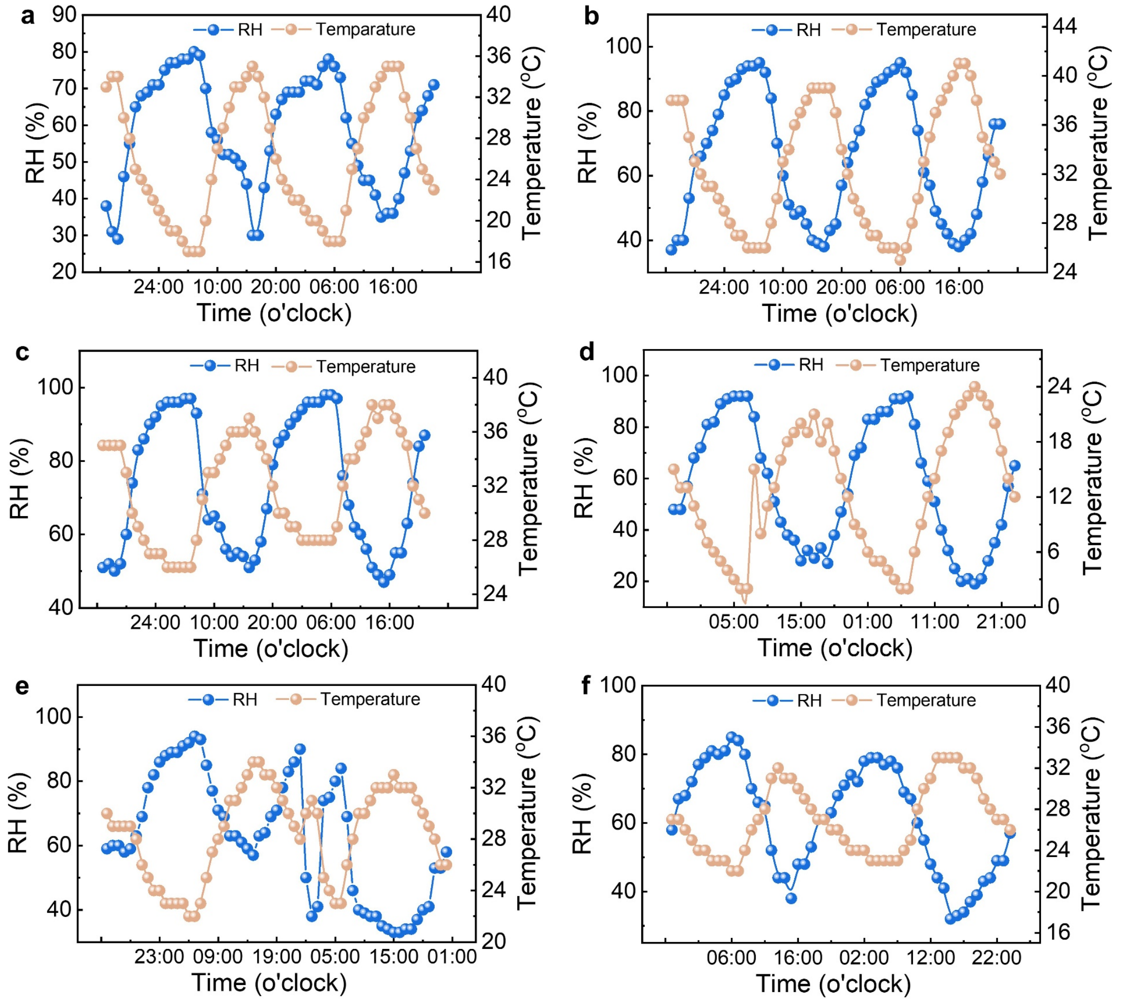

Supplementary Fig. 1 The variation of relative humidity (RH) and temperature in **a** Shennongjia, **b** Shiyan, **c** Wuhan, **d** Yushu, **e** Beijing, and **f** Guiyang (China, Date on July 30th to September 1st, 2021).

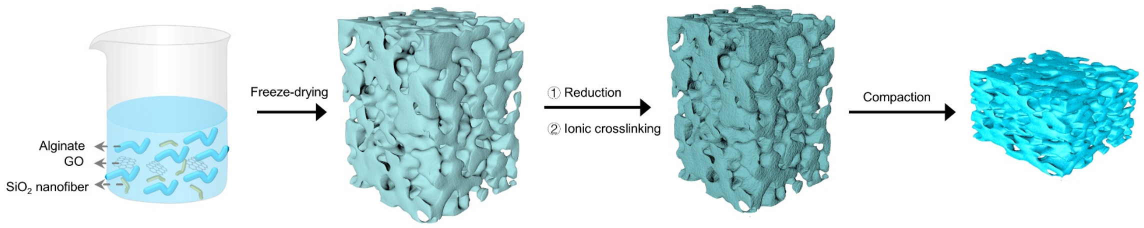

Supplementary Fig. 2 Schematic illustration of the preparation process of porous SAG film.

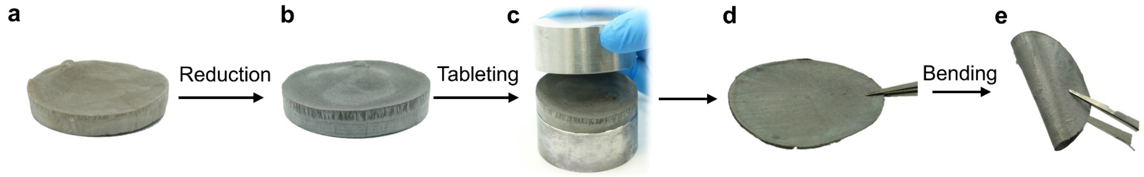

Supplementary Fig. 3 Photos of **a** freeze-dried aerogel, **b** chemical reduced aerogel, **c** the subsequent tableting treatment process under 10 MPa, **d** obtained SAG film, and **e** the bent SAG film.

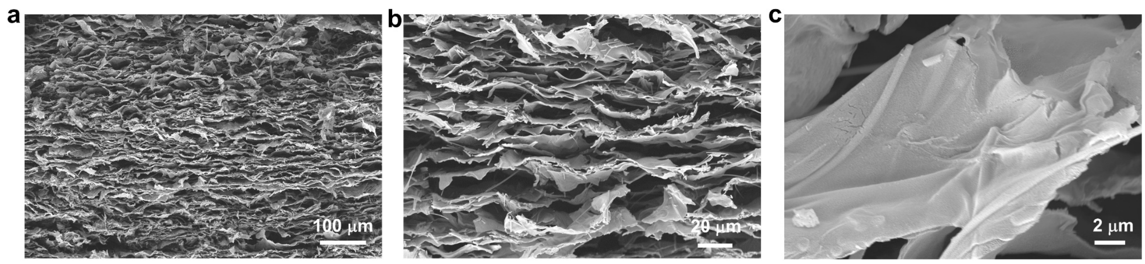

Supplementary Fig. 4 Cross-sectional SEM images of SAG film with different magnification of **a** 150 times, **b** 550 times, and **c** 5,000 times.

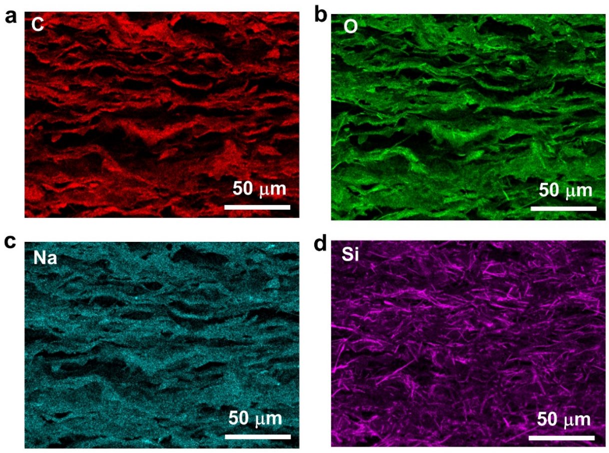

Supplementary Fig. 5 Element mapping images of **a** C, **b** O, **c** Na, and **d** Si elements, respectively, displaying the uniform combination of SA chains, SiO_2 nanofibers, and rGO nano-sheets.

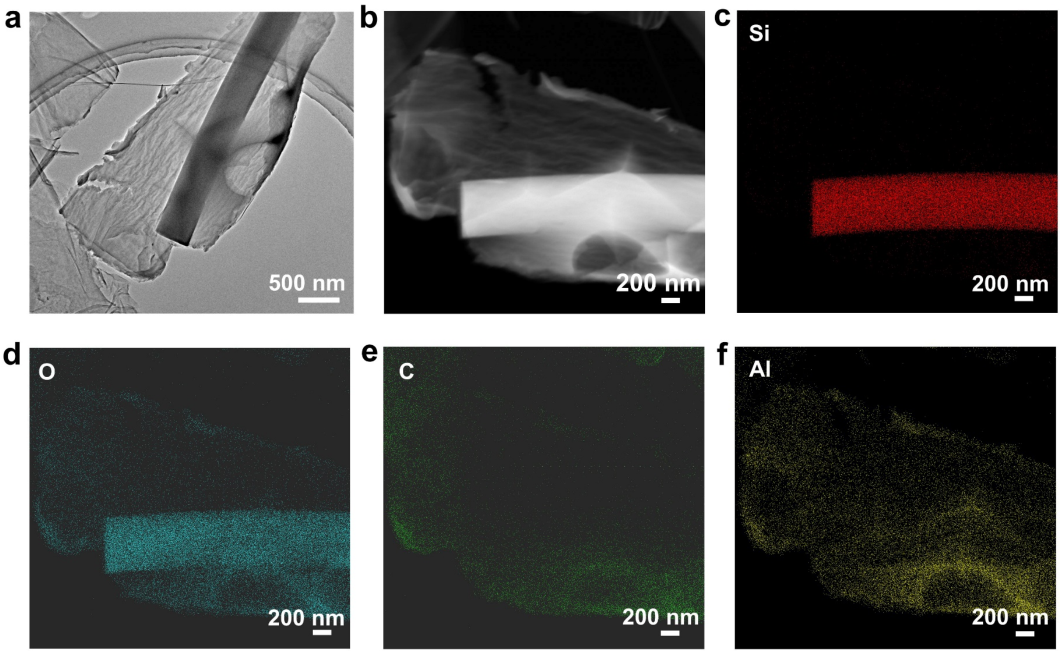

Supplementary Fig. 6 a,b TEM images of SAG film at different magnification. **c–f** Corresponding element mapping (Si, O, C, Al) images.

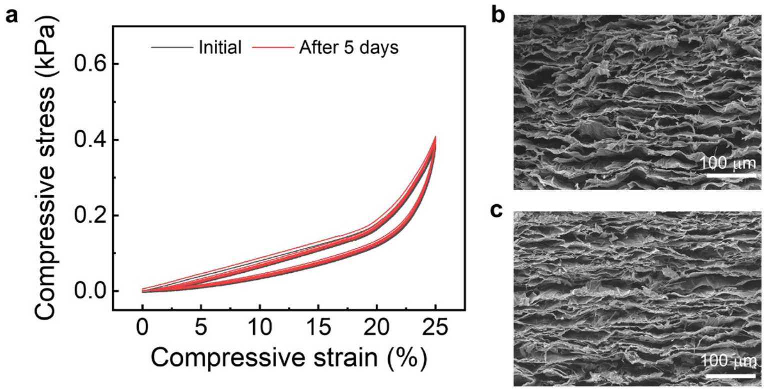

Supplementary Fig. 7 a Compressive stress versus compressive strain for SAG before and after 5 days immersing into water. SEM images for SAG film **b** before and **c** 5 days immersing in water.

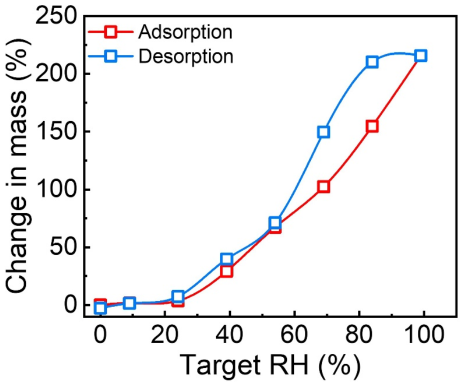

Supplementary Fig. 8 Moisture adsorption and desorption isotherm for SAG film at 40 ºC.

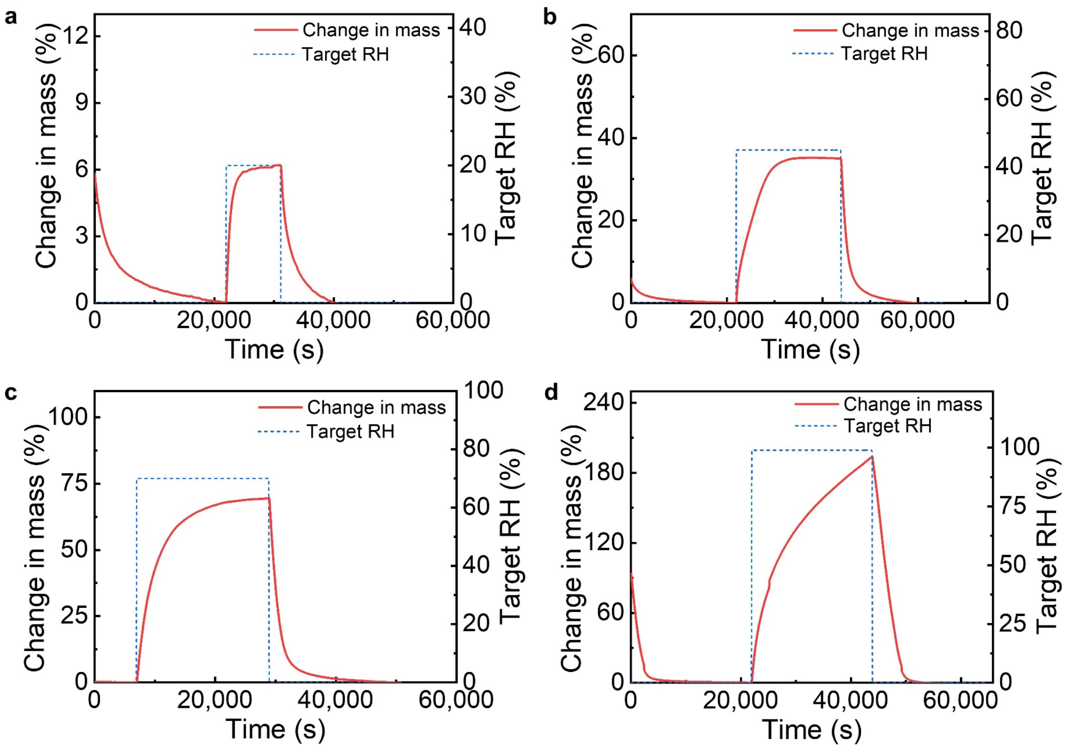

Supplementary Fig. 9 Moisture adsorption and desorption profiles under **a** 20% RH, **b** 45% RH, **c** 70% RH, and **d** 99% RH condition (40°C).

Supplementary Table 1 The linear fitting for diffusion coefficient under different RH based on Fick's second law.

RH		Fitting equation	R^2
20%	Adsorption	$y = 0.035x - 0.333$	0.99934
	Desorption	$y = -0.015 + 1.062$	0.99177
45%	Adsorption	$y = 0.0114x - 0.091$	0.99877
	Desorption	$y = -0.0226 + 1.217$	0.99353
70%	Adsorption	$y = 0.0143x - 0.168$	0.99611
	Desorption	$y = -0.0225x + 1.277$	0.9959
99%	Adsorption	$y = 0.0101x - 0.119$	0.9947
	Desorption	$y = -0.0129x + 1.186$	0.9729

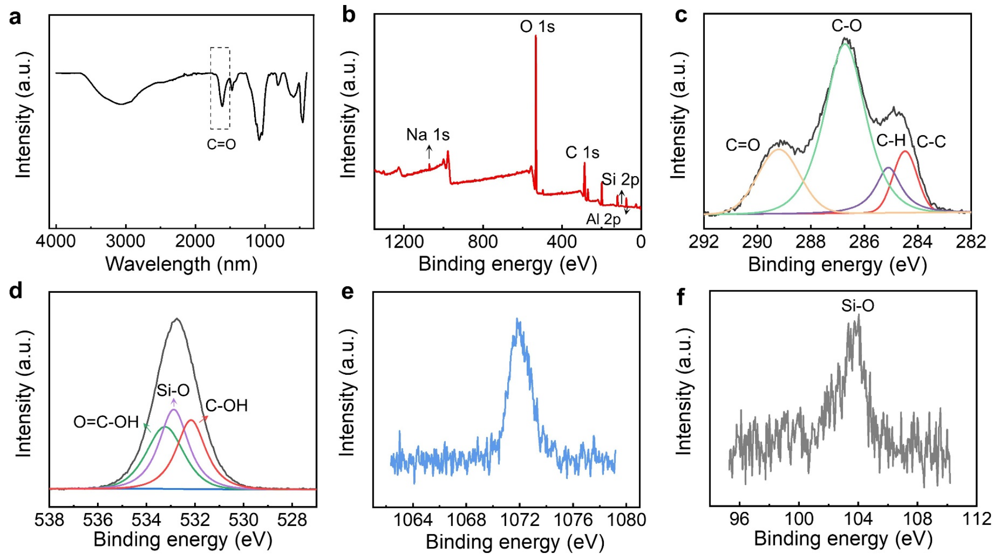

Supplementary Fig. 10 a FTIR spectrum of SAG film. The absorption peaks at 1612 cm^{-1} characteristic of symmetric stretching vibrations of carboxyl groups. **b** XPS survey spectrum of SAG film. **c–f** C 1s, O 1s, Na 1s, and Si 2p XPS spectra of SAG film.

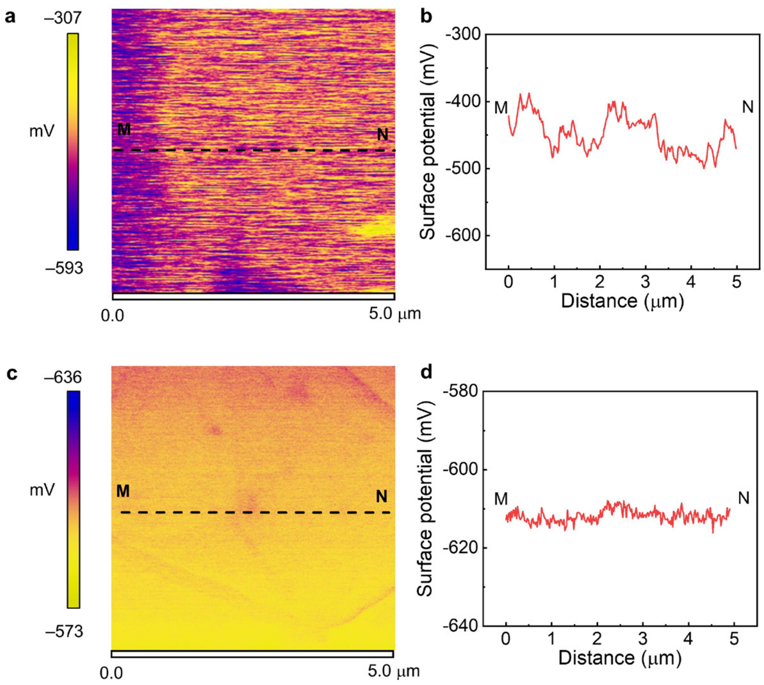

Supplementary Fig. 11 a KPFM images and **b** relative surface potential along the marked black line in 55% RH condition. **c** KPFM images and **d** relative surface potential along the marked black line in 100% RH condition.

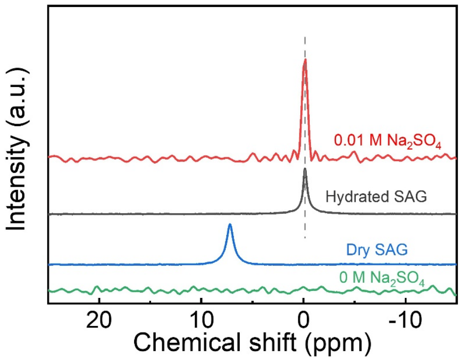

Supplementary Fig. 12 ^{23}Na solid state NMR spectra of 0.01 M Na_2SO_4 solution, hydrated SAG film, dry SAG film, and 0 M Na_2SO_4 solution. The ^{23}Na chemical shift of SAG film with water content of 167% is ~0 ppm, which accords with that of Na^+ ion of aqueous Na_2SO_4 solution. Furthermore, the Na^+ ions content (n/m_{dry}, n and m_{dry} denotes the mole of dissociated Na^+ ions and dry mass of SAG film, respectively) of SAG film could be obtained by calibration curve method.

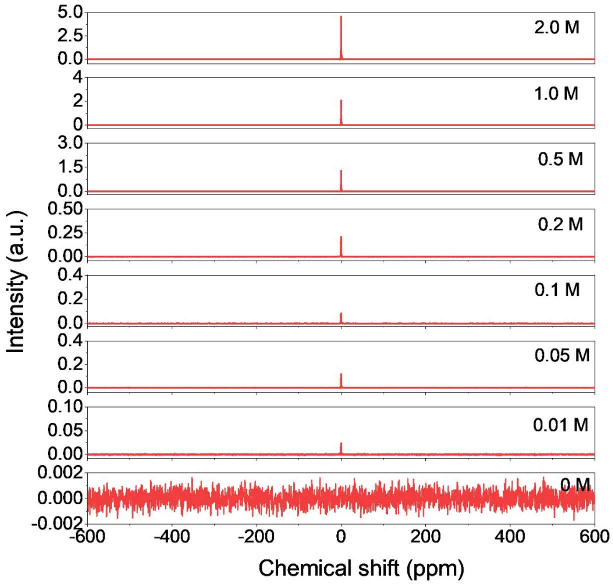

Supplementary Fig. 13 ^{23}Na solid state NMR spectra of Na_2SO_4 solution with various concentration.

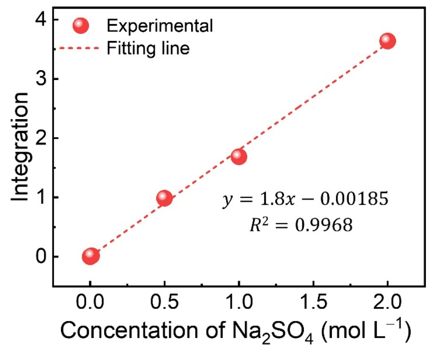

Supplementary Fig. 14 Plot of integration of characteristic peak in ^{23}Na NMR with different concentration of Na_2SO_4 solutions. By linear fitting, corresponding calibration curve was obtained.

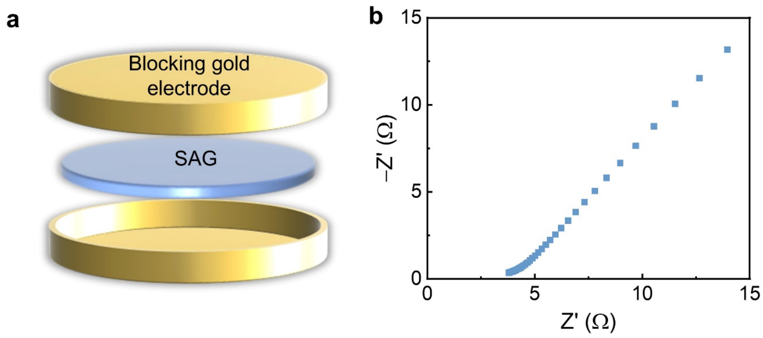

Supplementary Fig. 15 a Schematic of button-typed device for ionic conductance. The device was composed of a pair of gold electrodes as blocking electrodes and SAG film. **b** The enlarged electrochemical impedance spectra of SAG film at 100% RH.

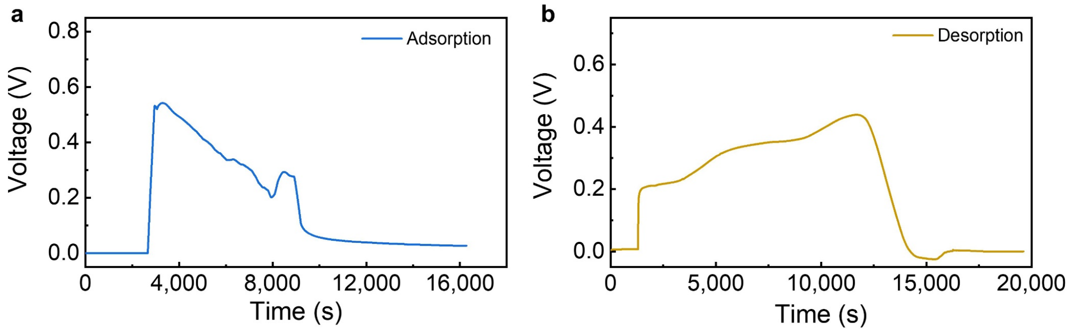

Supplementary Fig. 16 Voltage-time profiles in **a** moisture adsorption power generation and **b** desorption power generation.

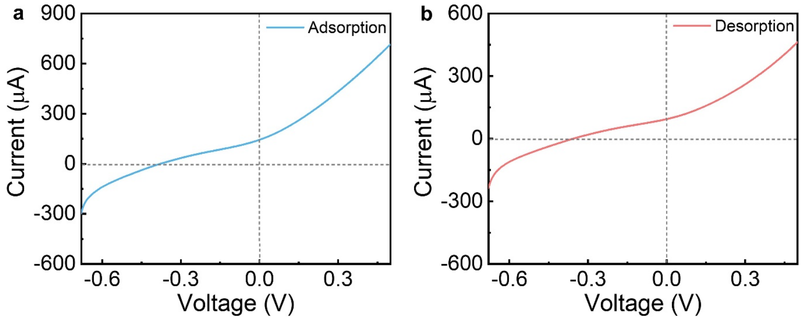

Supplementary Fig. 17 a Typical *I-V* curves from the MADG device at 100% RH and 40 °C. **b** Typical *I-V* curves from the MADG device at 15% RH and 40 °C.

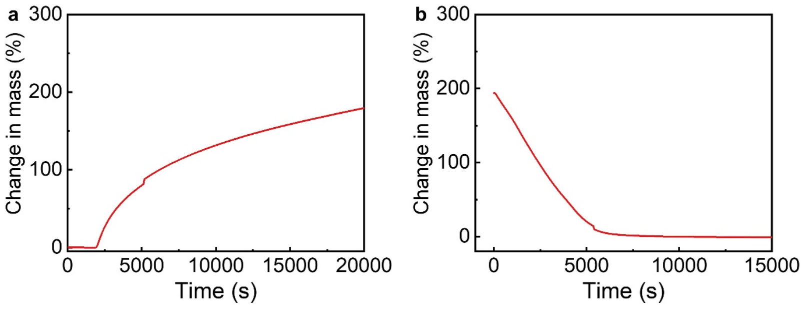

Supplementary Fig. 18 a The mass change of SAG film by water molecule adsorption versus time under ~100% RH and 40 °C. **b** The mass change of adsorption saturated SAG film by water molecule desorption versus time under 0% RH and 40 °C.

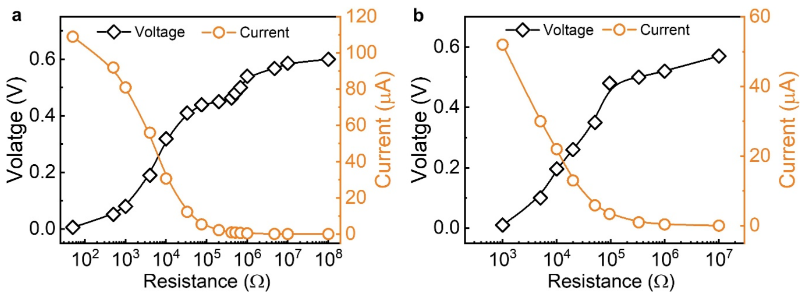

Supplementary Fig. 19 Voltage and current output of a MADG with varied electric resistances in **a** moisture-adsorbing power generation and **b** moisture-desorbing power generation.

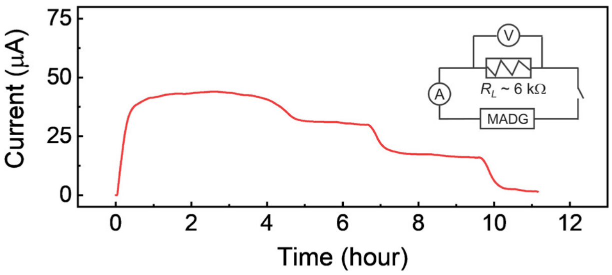

Supplementary Fig. 20 The current output supplied by MADG device connected with a resistor of 6 kΩ. Inset displays the schematic of circuit.

Supplementary Table 2 Summary of the resistances of commercial electronic device.

Device type	Power (W)	Resistance (Ω)
Table lamp	5	~260
Earphone		16~600
Electric fan	60	~800
Incandescent lamp	100	~500
Hot glue gun	120	~400
Electric blanket	150	~320
Infrared lamp	100	~484
Ultrasonic Cleaning Machine	80	~600

Supplementary Table 3 Output power density of water related power generators.

NO.	Material	Form of water	RH (%)	Area (cm^2)	Open circuit voltage (V)	Short circuit current (μA)	P_{max} ($mW\ m^{-2}$)	P_{output} ($mW\ m^{-2}$)	R (kΩ)	*Reference*
1	Biological nano-fibrous	Moisture	99	1.0	0.11	0.022	0.0242	0.0063	10,000	*11*
2	Ni-Al layered double hydroxide	Liquid water		6.0	0.6	0.3	0.3	0.075	2,000,000	*12*
3	Fabric coated with carbon black	Liquid water		27.0	0.53	3.91	0.77	0.094	63,000	*13*
4	Ni-Al layered double hydroxide	Liquid water		15	0.7	1.3	0.6	0.12	270,000	*14*
5	Printable carbon film	Liquid water		13.5	1.0	0.6	0.44	0.13	1,500,000	*15*
6	Graphene oxide membrane	Moisture	25	0.04	0.205	0.036	1.84	0.184	10,000,000	*16*
7	MoS_2 film	Moisture	70	1.0	0.019	6.24	1.18	0.3	3,000	*17*
8	Bilayer of polyelectrolyte film	Moisture	25	0.5	0.95	0.04	0.76	0.46	20,000,000	*18*
9	Graphene oxide and sodium polyacrylate	Moisture	80	0.1	0.6	0.12	7.2	0.7	10,000,000	*19*
10	Carbon-coated cotton fabric	Moisture	37	27.0	0.74	22.5	6.2	0.74	20,000	*20*
11	Gradient graphene oxide and graphene oxide	Moisture	80	0.16	1.52	0.136	12.92	1.11	10,000,000	*21*
12	Corn stalk	Moisture	75	3.0	0.56	3.6	6.72	1.77	741,100	*22*
13	Protein nanowires	Moisture	50	0.25	0.53	0.35	7.46	1.865	1,000,000	*23*
14	Carbon black and PVA		55	12	0.52	63	27.3	6.75	9,000	*24*
15	Pencil-paper	Moisture	95	0.87	0.48	8.0	44	8.5	40,000	*25*
16	Porous carbon black/PVA film		55	16	1.04	165	107	24.5	5,520	*26*
17	Nanostructured silicon	Moisture	85	0.02	0.6	2.0	600	79	750,000	*27*
18	Silicon nanowire arrays	Moisture	45	4.0	0.4	220	220	60	1,000	*28*
19	Poly(4-styrensulfonic acid) membrane	Moisture	80		0.8	150	1200	170	24,400	*29*
20	SA-SiO_2-rGO	Moisture	100	0.89	0.6	109	730	120	4,000	*This work*

Supplementary Table 4 Summary of output volumetric power density of water-related power generators.

NO.	Material	Volume (cm^3)	R ($k\Omega$)	P_{output} ($\mu W\ cm^{-3}$)	*Reference*
1	Biological nano-fibrous	0.2	10,000	0.003	*11*
2	Ni-Al layered double hydroxide	0.003	2,000,000	15	*12*
3	Fabric coated with carbon black	0.32	63,000	0.78	*13*
4	Ni-Al layered double hydroxide	0.011	270,000	16.1	*14*
5	Printable carbon film	0.021	1,500,000	8.1	*15*
6	Graphene oxide membrane	4.8×10^{-4}	10,000,000	1.5	*16*
7	MoS_2 film	5×10^{-4}	3,000	42	*17*
8	Bilayer of polyelectrolyte film	0.005	20,000,000	0.9	*18*
9	Graphene oxide and sodium polyacrylate	0.001	10,000,000	7	*19*
10	Carbon-coated cotton fabric	0.32	20,000	6.2	*20*
11	Gradient graphene oxide and graphene oxide	0.0016	10,000,000	11.1	*21*
12	Corn stalk	0.6	741,100	0.89	*22*
13	Protein nanowires	1.75×10^{-4}	1,000,000	266	*23*
14	Poly(4-styrensulfonic acid) membrane	0.02	24,400	850	*29*
15	SA-SiO_2-rGO	0.018	4,000	600	*This work*

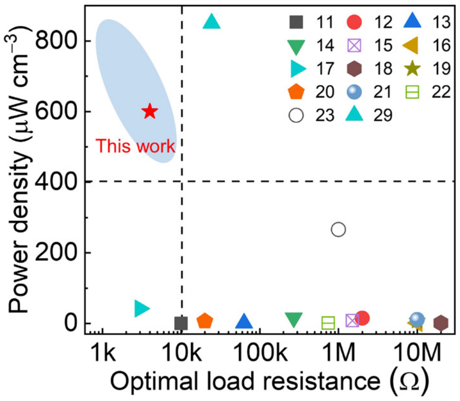

Supplementary Fig. 21 Comparison of output volumetric power density and internal resistance between MADG device in this work and reported water induced generators. It should be pointed that there are lack of descriptions of specific size of device in many reported papers. And most of the reported papers only represent the area or thickness of electricity-generating materials, and no information on size of electrodes. Thus, the mentioned output volumetric power density is calculated by the volume of electricity-generating material.

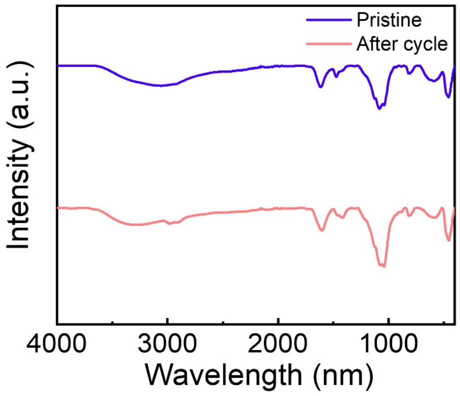

Supplementary Fig. 22 FTIR spectroscopy of the SAG film before and after 10 cycles electricity-generating test.

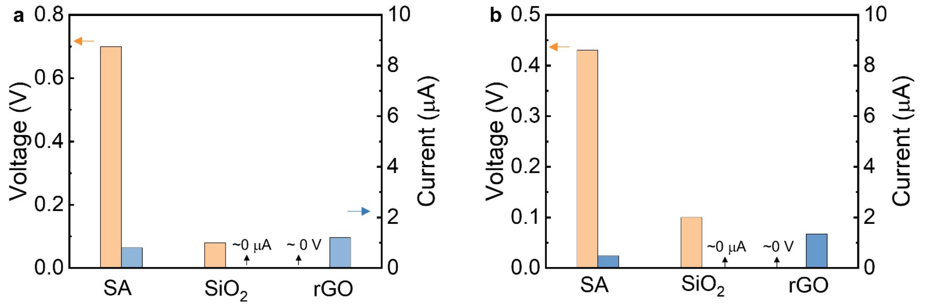

Supplementary Fig. 23 The generated open-circuit voltage and short-circuit current of the MADG device in **a** moisture adsorption and **b** desorption power generation.

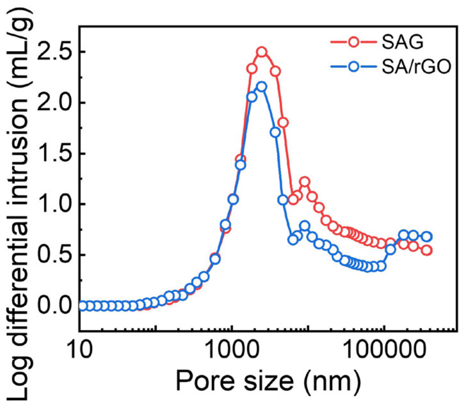

Supplementary Fig. 24 Pore size distribution for SAG and SA-rGO film by MIP testing. And the porosity of SAG and SA-rGO is 77% and 70%, respectively, demonstrating the SAG film possesses superior porosity.

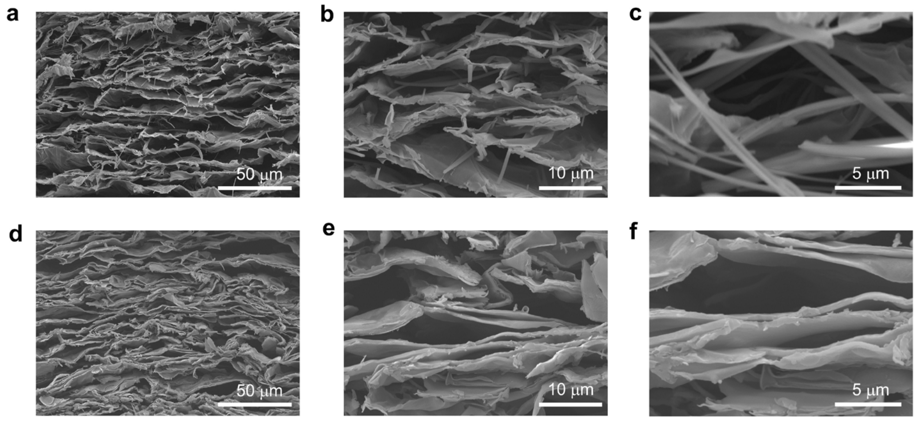

Supplementary Fig. 25 a–c SEM images at different magnification for SAG film and **d–f** SA-rGO film.

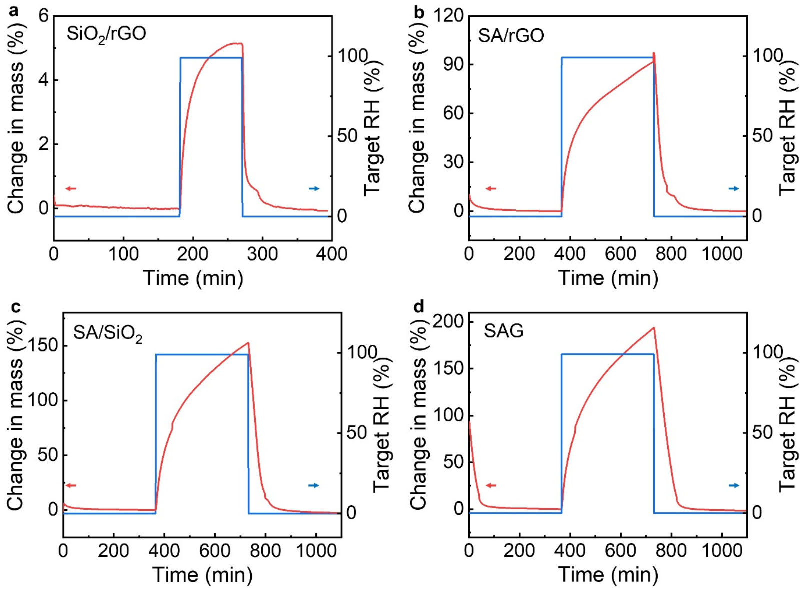

Supplementary Fig. 26 Kinetic water sorption profile for **a** SiO_2/rGO, **b** SA/rGO, **c** SA/SiO_2, and **d** SAG film. The testing samples were first equilibrated to 0% RH, then equilibrated to ~100% RH (adsorption step), and then equilibrated back to 0% RH (desorption step) again, with all steps being carried out at 40 °C.

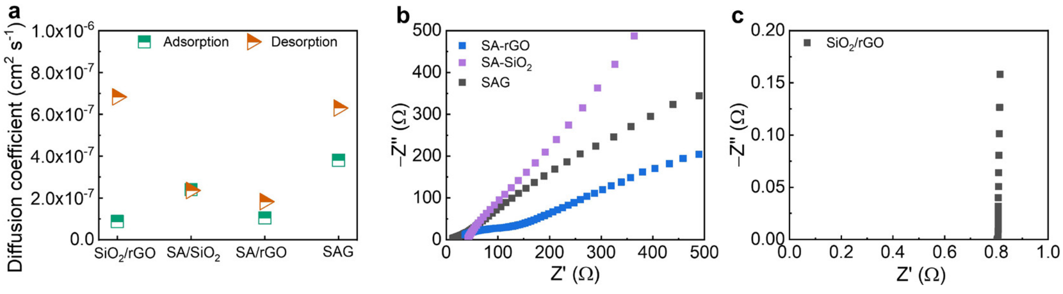

Supplementary Fig. 27 a Adsorption and desorption diffusion coefficients of SiO_2/rGO, SA/SiO_2, SA/rGO, and SAG film at ~100% RH and 40 °C. **b,c** The electrochemical impedance spectra of SiO_2/rGO, SA/SiO_2, SA/rGO, and SAG film at 100% RH.

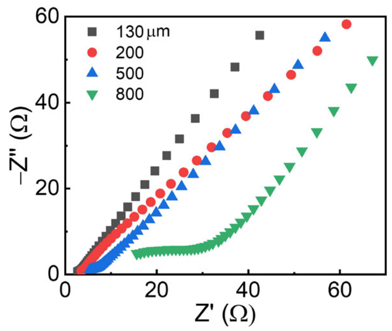

Supplementary Fig. 28 The electrochemical impedance spectra of SAG film with different thickness at 100% RH.

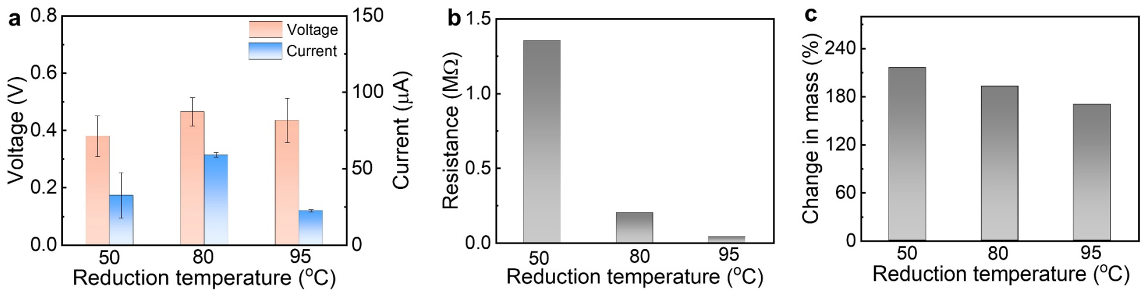

Supplementary Fig. 29 a Voltage and current output for moisture-desorbing power generation based on SAG film with various reduction temperature. Error bars represent the standard deviations from multiple measurements. **b** Resistance variation of dry MADG with different reduction temperature. **c** The mass change of SAG film with various reduction reduction at 100% RH.

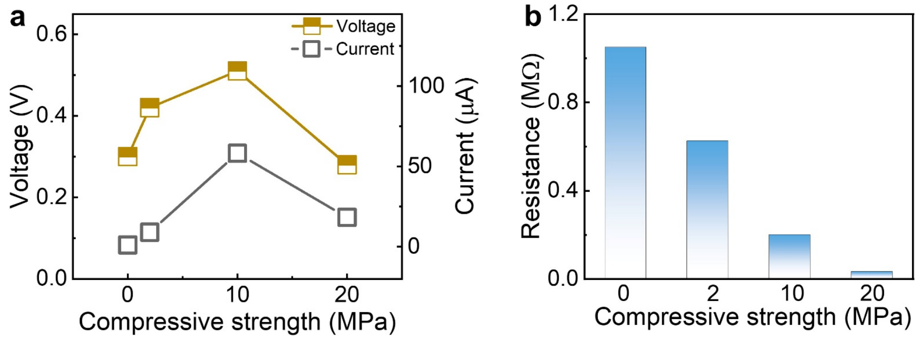

Supplementary Fig. 30 a Voltage and current output versus the compressive strength of SAG film for moisture-desorbing power generation (15±5% RH, 35±5 ºC). **b** The resistance of dry MADG device with different compressive strength of SAG film.

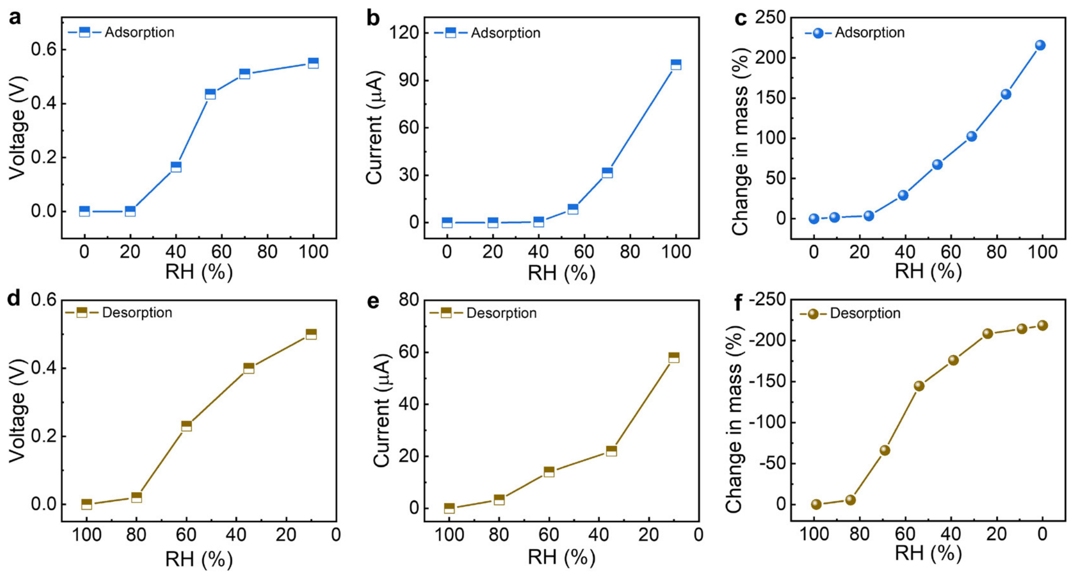

Supplementary Fig. 31 a,b Voltage and current output of device in response to different RH during moisture adsorption power generation (40ºC, initial RH of 0%). **c** The mass change of SAG film by water molecule adsorption upon different RH under 40ºC. **d,e** Voltage and current output of hydrated device in response to different RH during moisture desorption power generation (40ºC, initial RH of 100%). **f** The mass change of saturated SAG film by water molecule adsorption upon different RH under 40ºC.

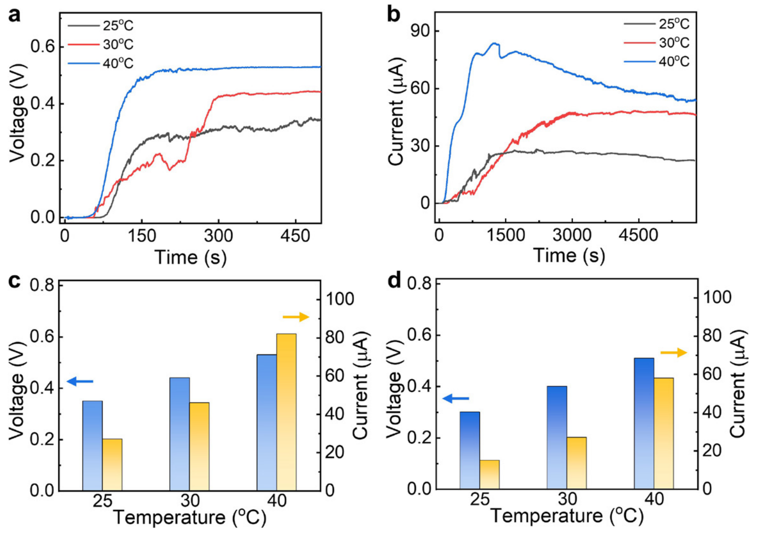

Supplementary Fig. 32 a The voltage-time and **b** current-time profile in moisture adsorption electricity generation under different temperature (~100% RH). **c** The generated voltage and current in moisture adsorption power generation and **d** desorption power generation under different temperature.

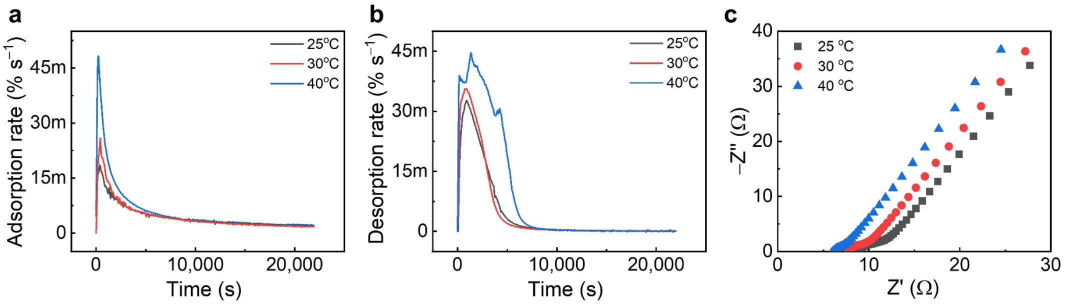

Supplementary Fig. 33 a Rate of adsorbing water molecules during moisture adsorption under different temperature and ~100% RH. **b** Rate of desorbing water molecules during moisture desorption under different temperature and ~15% RH. **c** The electrochemical impedance spectra of MADG device under different temperature and ~100% RH.

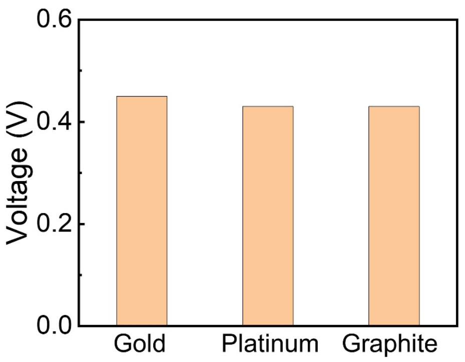

Supplementary Fig. 34 The induced voltage of the MADGs with different electrodes, including gold, platinum, and graphite electrodes.

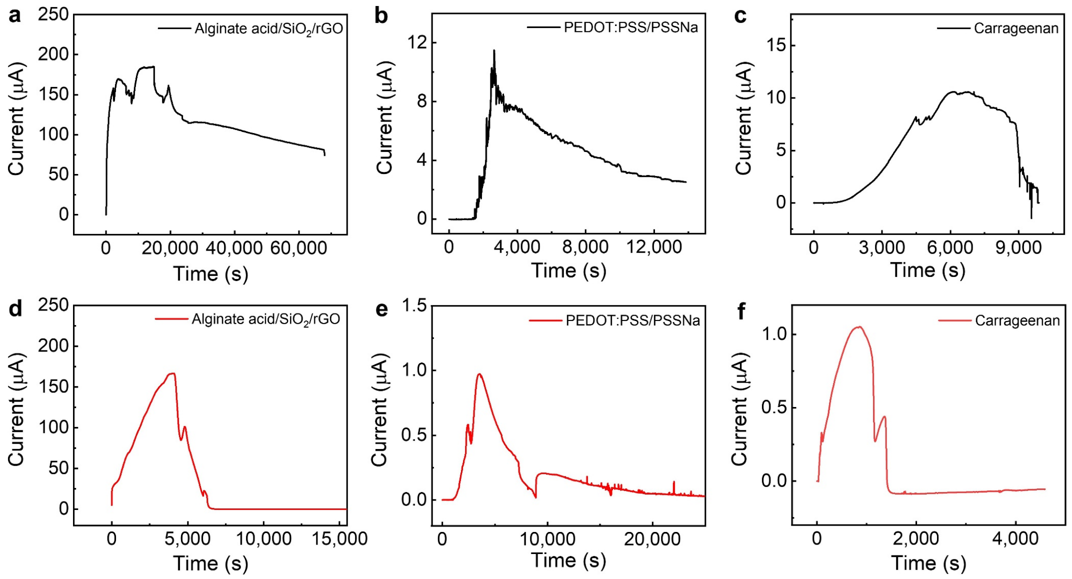

Supplementary Fig. 35 Short-circuit current generated by **a** alginate acid/SiO_2/rGO, **b** PEDOT: PSS/PSSNa, and **c** carrageenan film during moisture adsorption power generation. Short-circuit current generated by **d** alginate acid/SiO_2/rGO, **e** PEDOT: PSS/PSSNa, and **f** carrageenan film during moisture desorption electricity generation.

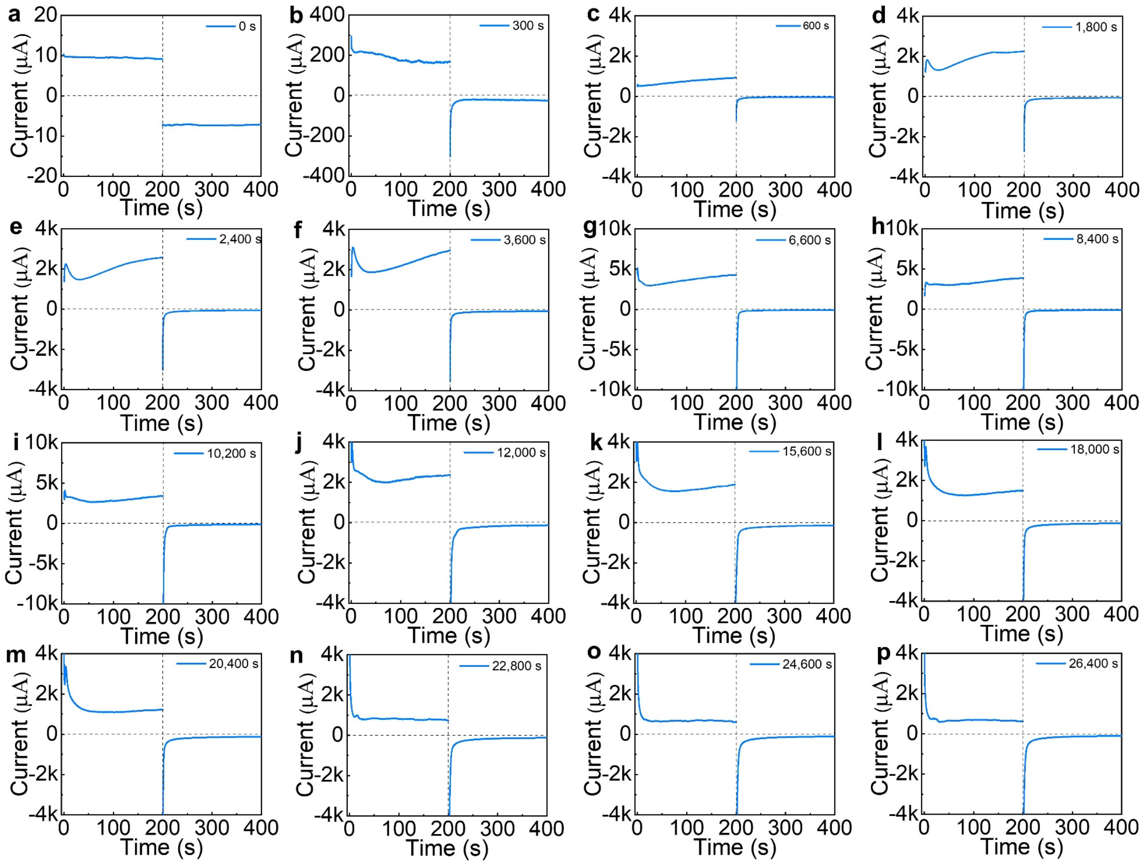

Supplementary Fig. 36 Current responses curves of MADG device under alternating bias (± 1 V) during adsorption process.

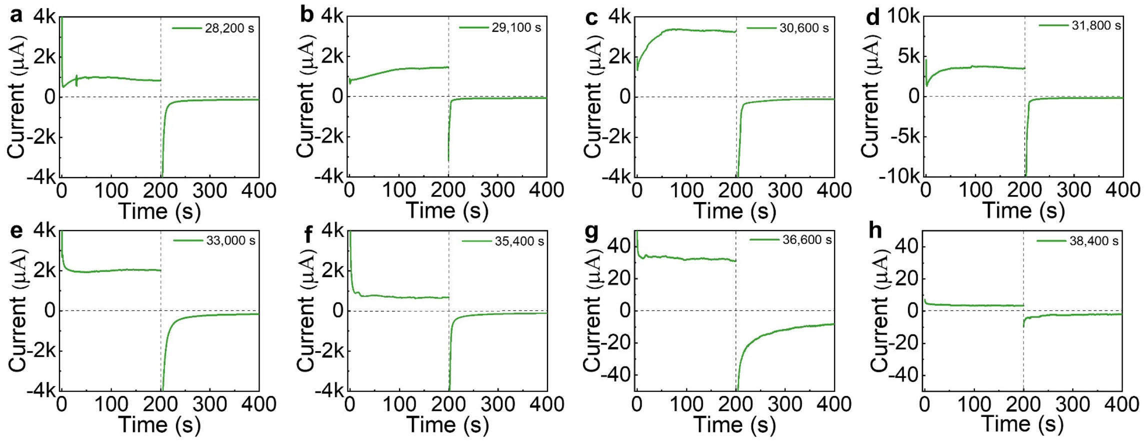

Supplementary Fig. 37 Current responses curves of MADG device under alternating bias (± 1 V) during adsorption process.

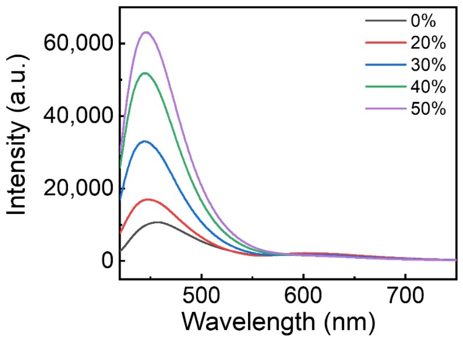

Supplementary Fig. 38 Emission spectra of fluorescence probe response to different water content in ethanol under excitation at 407 nm.

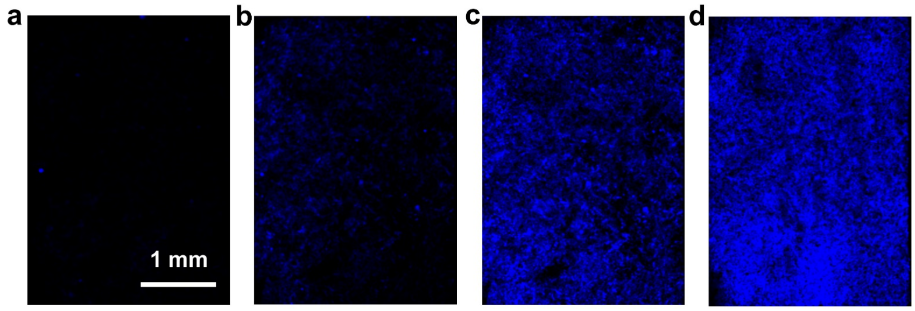

Supplementary Fig. 39 Surface CLSM images during adsorption process for **a** 0 min, **b** 50 min, **c** 70 min, and **d** 370 min. The blue fluorescence distribution represents the water distribution.

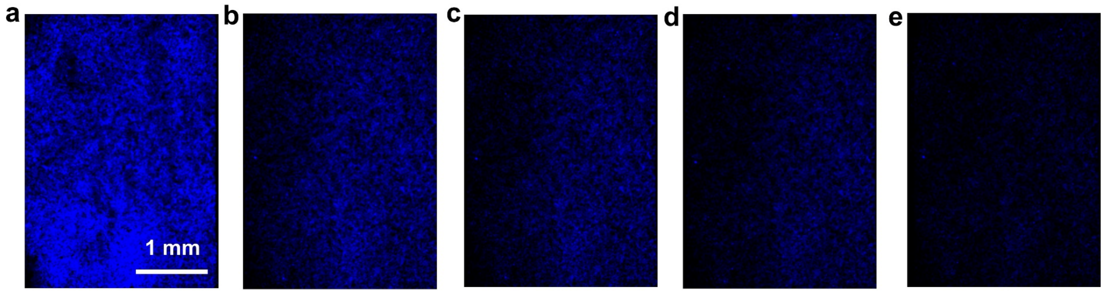

Supplementary Fig. 40 Surface CLSM images during desorption process for **a** 0 min, **b** 90 min, **c** 110 min, **d** 130 min, and **e** 1370 min.

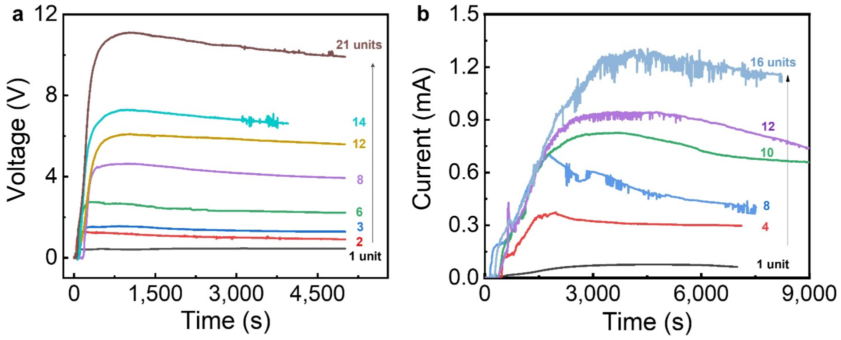

Supplementary Fig. 41 a The supplied voltage signals of integrated device with 1, 2, 3, 6, 8, 12, 14, and 21 units in series. **b** The current signals of integrated device with 1, 4, 8, 10, 12, and 16 units in parallel connection.

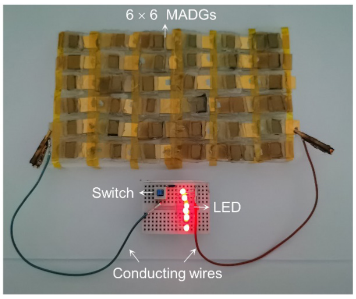

Supplementary Fig. 42 Six red LEDs directly powered by 6 × 6 integrated MADG device.

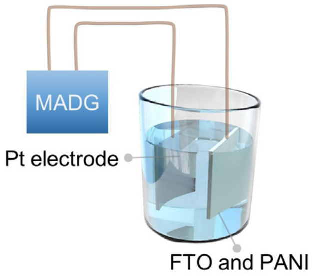

Supplementary Fig. 43 Schematics of the MADG-driven electrochemical polymerization or electrochromic of polyaniline (PANI).

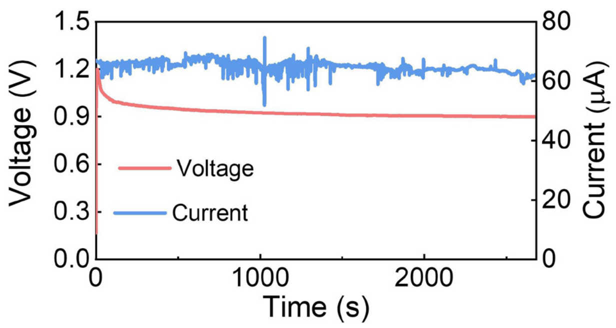

Supplementary Fig. 44 Schematics of the MADG-driven electrochemical polymerization or electrochromic of polyaniline (PANI).

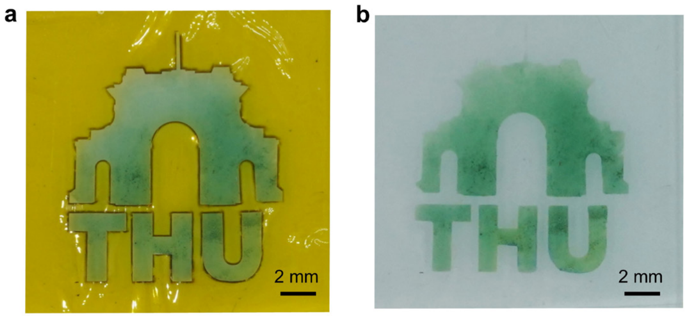

Supplementary Fig. 45 Digital photos of patterned PANI film with **a** mask and **b** without mask.

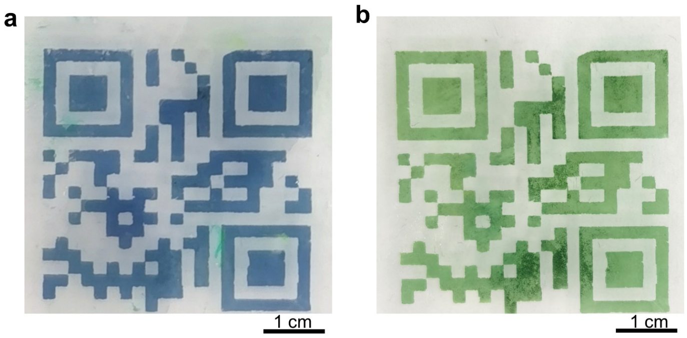

Supplementary Fig. 46 The photos of PANI film with dimensional barcode pattern under **a** un-doped state and **b** doped state.

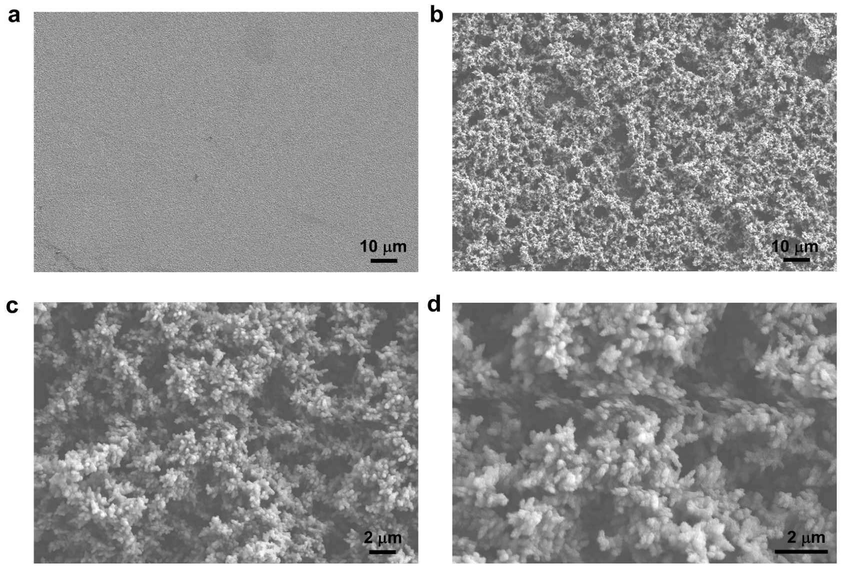

Supplementary Fig. 47 **a** SEM image of fluorine doped tin oxide (FTO) substrate. **b–d** SEM images of the electro-polymerized PANI film on FTO at different magnifications. The electrochemical polymerization was powered by MADG device.

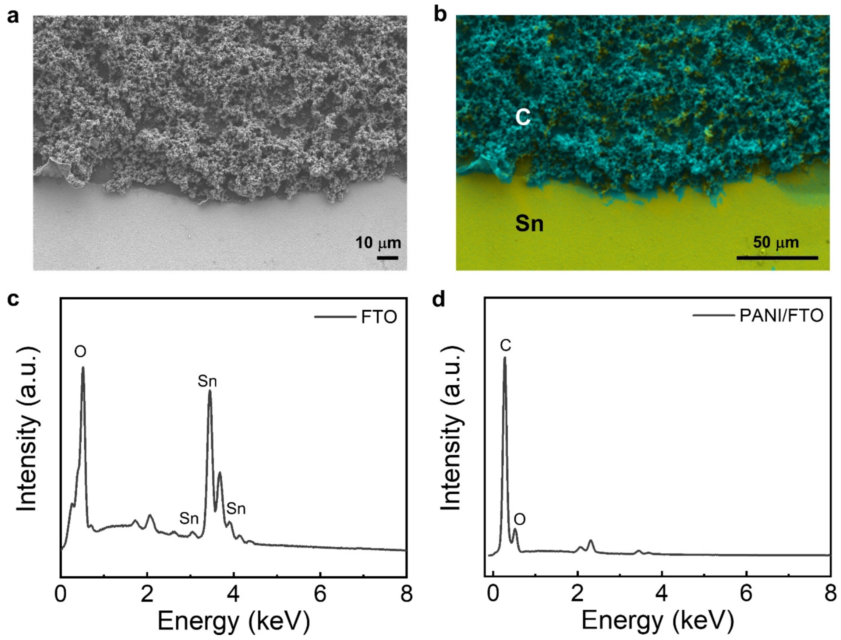

Supplementary Fig. 48 **a** SEM image of PANI film by electrochemical polymerization driven by MADG. **b** Element mapping image of the C element from PANI component, and the Sn

element from FTO conductive substrate. EDS of **c** FTO substrate and **d** electro-polymerized PANI film.

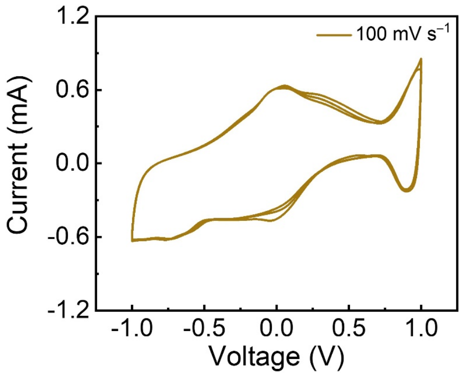

Supplementary Fig. 49 Cyclic voltammetry curve of the obtained PANI film performed in 1 M H_2SO_4 aqueous solution at the scan rate of 100 mV s^{-1}.

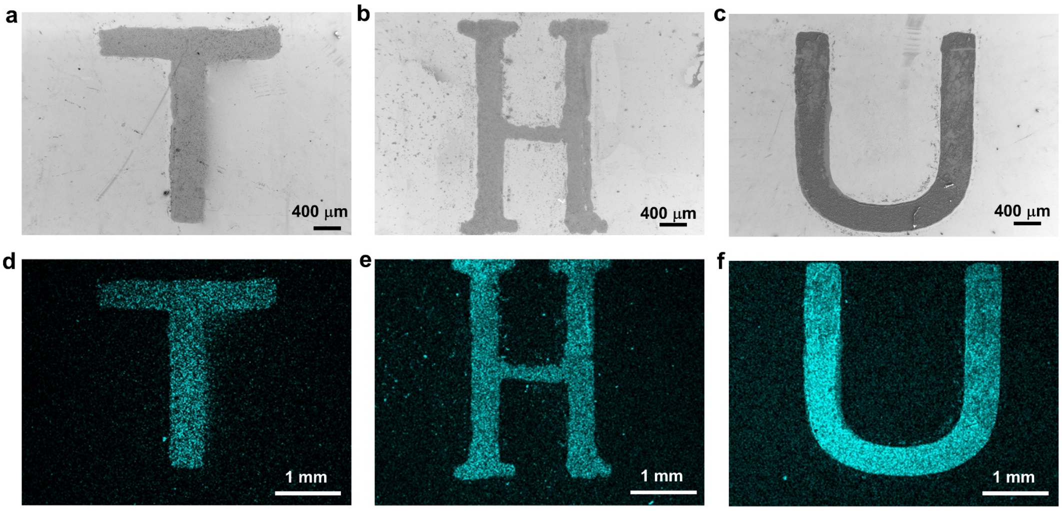

Supplementary Fig. 50 a–c Optical microscope images of PANI film with "THU" patterns. **d–f** Corresponding EDS images of PANI film with "THU" patterns.

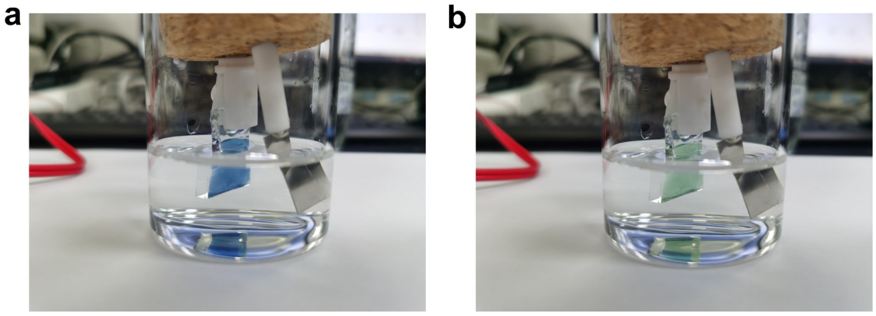

Supplementary Fig. 51 a Photograph of electrochromic apparatus during oxidation process. **b** Photo of electrochromic apparatus during reduction process.

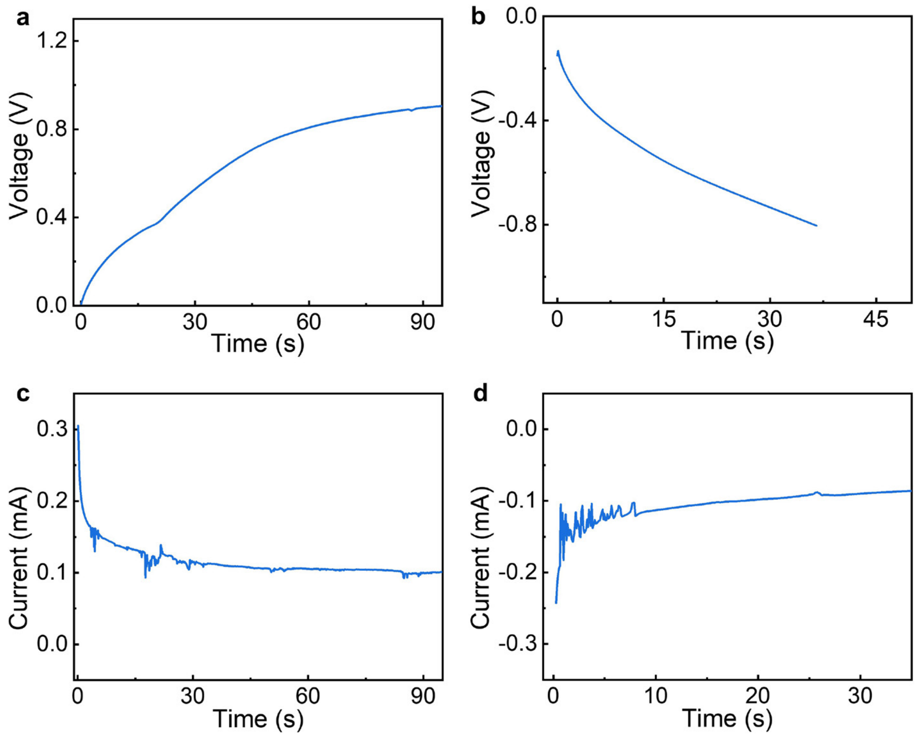

Supplementary Fig. 52 Voltage and Current between the two electrodes in solution during electrochromic. Voltage-time curves were recorded from **a** oxidation process and **b** reduction process. Current-time curves were originated from **c** oxidation process and **d** reduction process.

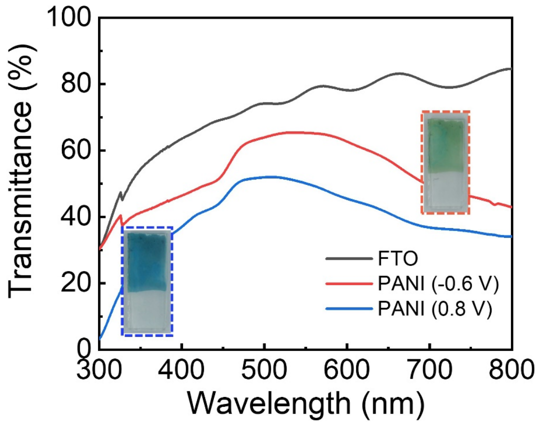

Supplementary Fig. 53 Transmittance spectra of FTO glass and the PANI film on FTO under different voltage conditions, and the insets are the corresponding digital photos of the PANI film.

References

1 Berendsen, H. J. C., Grigera, J. R. & Straatsma, T. P. The missing term in effective pair potentials. *J. Phys. Chem.* **91**, 6269–6271 (1987).

2 Straatsma, T. P. & Berendsen, H. J. C. Free energy of ionic hydration: analysis of a thermodynamic integration technique to evaluate free energy differences by molecular dynamics simulations. *J. Chem. Phys.* **89**, 5876–5886 (1988).

3 Xiong, W. et al. Strain engineering water transport in graphene nanochannels. *Phys. Rev. E* **84**, 056329 (2011).

4 Muscatello, J., Jaeger, F., Matar, O. K. & Müller, E. A. Optimizing water transport through graphene-based membranes: insights from nonequilibrium molecular dynamics. *ACS Appl. Mater. Interfaces* **8**, 12330–12336 (2016).

5 Stuart, S. J., Tutein, A. B. & Harrison, J. A. A reactive potential for hydrocarbons with intermolecular interactions. *J. Chem. Phys.* **112**, 6472–6486 (2000).

6 Plimpton, S. Fast parallel algorithms for short-range molecular dynamics. *J. Comput. Phys.* **117**, 1–19 (1995).

7 Cranford, S. & Buehler, M. J. Twisted and coiled ultralong multilayer graphene ribbons. *Modell. Simul. Mater. Sci. Eng.* **19**, 054003 (2011).

8 Battaile, C. C. The kinetic Monte Carlo method: foundation, implementation, and application. *Comput. Methods Appl. Mech. Engrg.* **197**, 3386–3398 (2008).

9 Bortz, A. B., Kalos, M. H. & Lebowitz, J. L. A new algorithm for Monte Carlo simulation of Ising spin systems. *J. Comput. Phys*. **17**, 10–18 (1975).

10 Gillespie, D. T. Exact stochastic simulation of coupled chemical reactions. *J. Phys. Chem.* **81**, 2340–2361 (1977).

11 Li, M. J. et al. Biological nanofbrous generator for electricity harvest from moist air flow. *Adv. Funct. Mater.* **29**, 1901798 (2019).

12 Tian, J. L. et al. Surface charge density-dependent performance of Ni–Al layered double hydroxide-based flexible self-powered generators driven by natural water evaporation. *Nano Energy* **70**, 104502 (2020).

13 Yun, T. G., Bae, J., Rothschild, A. & Kim, I.-D. Transpiration driven electrokinetic power generator. *ACS Nano* **13**, 12703−12709 (2019).

14 Sun, J. C. et al. Electricity generation from a Ni-Al layered double hydroxide-based flexible generator driven by natural water evaporation. *Nano Energy* **57**, 269−278 (2019).

15 Ding, T. P. et al. All-printed porous carbon film for electricity generation from evaporation-driven water flow. *Adv. Funct. Mater.* **27**, 1700551 (2017).

16 Cheng, H. H. et al. Spontaneous power source in ambient air of a well-directionally reduced graphene oxide bulk. *Energy Environ. Sci.* **11**, 2839–2845 (2018).

17 He, D. R. et al. Electricity generation from phase-engineered flexible MoS2 nanosheets under moisture. *Nano Energy* **81**, 105630 (2021).

18 Wang, H. Y. et al. Bilayer of polyelectrolyte films for spontaneous power generation in air up to an integrated 1,000 V output. *Nat. Nanotechnol.* **16**, 811–819 (2021).

19 Huang, Y. X. et al. All-region-applicable, continuous power supply of graphene oxide composite. *Energy Environ. Sci.* **12**, 1848–1856 (2019).

20 Bae, J., Yun, T. G., Suh, B. L., Kim, J. & Kim, I.-D. Self-operating transpiration-driven electrokinetic power generator with an artificial hydrological cycle. *Energy Environ. Sci.* **13**, 527–534 (2020).

21 Huang, Y. X. et al. Interface-mediated hygroelectric generator with an output voltage approaching 1.5 volts. *Nat. Commun.* **9**, 4166 (2018).

22 Gong, F. et al. Agricultural waste-derived moisture-absorber for all-weather atmospheric water collection and electricity generation. *Nano Energy* **74**, 104922 (2020).

23 Liu, X. M. et al. Power generation from ambient humidityusing protein nanowires. *Nature* **578**, 550–554 (2020).

24 Li, L. H. et al. Sustainable and flexible hydrovoltaic power generator for wearable sensing electronics. *Nano Energy* **72**, 104663 (2020).

25 Xu, Y. D. et al. Pencil–paper on-skin electronics. *Proc. Natl. Acad. Sci. USA* **117**, 18292–18301 (2020).

26 Li, L. H. et al. A novel, flexible dual-mode power generator adapted for wide dynamic range of the aqueous salinity. *Nano Energy* **85**, 105970 (2021).

27 Shao, B. B. et al. Bioinspired hierarchical nanofabric electrode for silicon hydrovoltaic device with record power output. *ACS Nano* **15**, 7472−7481 (2021).

28 Qin, Y. S. et al. Constant electricity generation in nanostructured silicon by evaporation-driven water flow. *Angew. Chem. Int. Ed.* **59**, 10619–10625 (2020).

29 Xu,T. et al. An efficient polymer moist-electric generator. *Energy Environ. Sci.* **12**, 972–978 (2019).
