# Supplementary material for: Moisture adsorption-desorption full cycle power generation
Source: Nat Commun. 2022 May 9;13:2524. doi: 10.1038/s41467-022-30156-3 (PMC9085775; doi:10.1038/s41467-022-30156-3)
Supplement: Supplementary file 3 — Description of Additional Supplementary Files [file 41467_2022_30156_MOESM3_ESM.pdf]

Description of Additional Supplementary Files

File Name: Supplementary Movie 1
Description: LED bulb directly powered by MADGs.
